# Recovery with a fan-cooling jacket after exposure to high solar radiation during exercise in hot outdoor environments

**DOI:** 10.3389/fspor.2023.1106882

**Published:** 2023-02-13

**Authors:** Takashi Naito, Tatsuya Saito, Mitsunori Ohhashi, Sotaro Hayashi

**Affiliations:** ^1^ Faculty of Law, Hokkai-Gakuen University, Sapporo, Japan; ^2^ Faculty of Medicine, Tottori University, Tottori, Japan; ^3^ Faculty of Human Health, Kurume University, Kurume, Japan; ^4^ Faculty of Urban Management, Fukuyama City University, Fukuyama, Japan

**Keywords:** fan-cooling condition, clear sky, rectal temperature, hyperthermia, tympanic temperature

## Abstract

The study aimed to investigate the effect of body cooling with a fan-cooling jacket on body temperature responses during recovery after exercise when exposed to high solar radiation in a hot outdoor environment. Nine males cycled using ergometer until their rectal temperature increased to 38.50 °C in hot outdoor environments, followed by body cooling recovery in warm indoor environments. Subjects repeatedly performed the cycling exercise protocol, which consisted of one set of 5 min at a load of 1.5 watt/kg body weight and 15 min at a load of 2.0 watt/kg body weight at 60 rpm. Body cooling recovery consisted of cold water ingestion (10°C: CON) or cold water ingestion + wearing a fan-cooling jacket (FAN) until the rectal temperature decreased to 37.75°C. The time for the rectal temperature to reach 38.5°C did not differ between the two trials. The rate of decrease in rectal temperature at recovery tended to be higher in FAN trial than in CON trial (*P* = 0.082). The rate of decrease in tympanic temperature was higher in FAN trials than in CON trials (*P* = 0.002). The rate of decrease in mean skin temperature at the first 20 min of recovery was higher in FAN than in CON trial (*P* = 0.013). Body cooling recovery with a fan-cooling jacket in addition to cold water ingestion may be effective in reducing elevated tympanic and skin temperatures after exercise in the heat under a clear sky, but may be difficult to decrease rectal temperature.

## Introduction

Due to its simplicity and ease of interpretation, Wet-bulb globe temperature (WBGT) is the most often used assessment of climatic heat stress. WBGT comprises of wet-bulb temperature, dry-bulb temperature, and black-globe temperature. Wet-bulb temperature is a measure of the efficiency of evaporation within the environment; as relative humidity (RH) decreases and/or air movement increases the wet-bulb temperature decreases. Dry-bulb temperature is a measure of the ambient temperature (Ta) within the environment. Black-globe temperature is a measure of radiant heat load, increasing solar radiation heats the black globe while increased air movement serves to cool the black globe. In the WBGT index equation, wet-bulb temperature, dry-bulb temperature, and black-globe temperature account for 70%, 10%, and 20% of the heat stress, respectively (1). Despite field training and major sporting competitions (e.g., Tokyo 2020, FIFA World Cup Qatar 2022) being conducted in hot outdoor environments (in the presence of solar radiation) (2, 3), the vast majority of scientific studies are conducted in indoor environments (4), without the presence of solar radiation.

Recently, studies have examined the effect of solar radiation levels on changes in the core temperature (Tc) and skin temperature during exercise in hot outdoor environments. A higher level of solar radiation, under clear-sky conditions increases mean skin temperature (Tsk) (2, 5, 6) during moderate-intensity exercise, as well as rectal temperature (Tre) during high-intensity interval training (7), which in turn impairs exercise capacity. Increased Tsk causes a reduction in the temperature gradient between the body and skin, which leads to a decreased ability to transfer heat to the environment (8), and sustained hyperthermia after exercise (9). Without appropriate cooling during recovery, the risk for hyperthermia and heat-related illnesses increases. Therefore, strategies to prevent hyperthermia, in hot outdoor environments, such as post-exercise cooling, are required.

Cold water immersion is the best strategy for reducing body temperature during body cooling recovery at post-exercise (10). Previous systematic reviews have shown that cold water immersion can rapidly reduce both Tc and Tsk to prevent heat stroke from exercise-induced hyperthermia (11, 12). However, cold water immersion typically requires a large tub filled with water and ice to be available for on-site use. When recreational athletes have limited to access to ample amounts of water and ice, or there is a lack of electricity to maintain water temperature, cooling may be considered using a different method. Recently, a fan-cooling jacket was recently developed to mitigate the thermal strain and the risk of heat stroke during activity in hot outdoor environments (13, 14). This jacket can be cooled *via* the process of circulating airflow underneath clothing by two small fans attached to the back of the waist and can be easily applied to recreational athletes. Post-exercise fanning in compensable heat stress has been proven to be an effective method to reduce the increased Tc and Tsk, accompanied by greater evaporative heat loss (15). Several previous studies have observed that fan-cooling jackets suppress the increased in both Tc and Tsk in hot indoor environments (16, 17). However, its application has not been studied during recovery from hyperthermia in post-exercise, and it is unclear whether it reduces Tc and Tsk more rapidly, which are elevated by exercise under high solar radiation.

This study aimed to investigate the effect of body cooling with a fan-cooling jacket on body temperature responses during recovery after exercising in hot outdoor environment with high solar radiation exposure. It was hypothesized that the fan cooling jacket would eliminate the increase in both Tc and Tsk caused due to high solar radiation exposure more rapidly.

## Methods

### Participants

Nine physically active males [age; 21 ± 1 years, height; 170.9 cm ± 5.0 cm, body mass (BM); 64.73 ± 6.38 kg,] were recruited for this study. Participants completed a minimum of 6 h of training per week at the time of study enrollment. All participants were non-smokers, and normotensive, had no known autonomic dysfunction or cardiovascular disease, and were not on any medications. The study protocol was approved by the Ethics Committees of Kurume (2021003) and Fukuyama City University (2021429) in Japan. All participants provided written informed consent before the commencement of the study. The study complied with the latest version of the Declaration of Helsinki and was conducted according to international standards.

### Experimental design

Participants performed two different trials during the post-exercise recovery period in a counterbalanced order as follows: cold water ingestion (CON) or wearing a fan-cooling jacket in addition to cold water ingestion (FAN). Before the experimental trials were initiated, participants underwent practice trials in a hot outdoor environment in order to familiarize them with the experimental protocol. This trial was identical to the experimental trials in all respects. All experimental trials were separated by at least 4 days to avoid an order effect. Throughout the study period, all participants were asked to keep their normal lifestyle activities at a stable level, including their physical activity and nutritional habits. During the 24-h period before the experimental trial, the participants were instructed to avoid strenuous exercise and the consumption of alcohol, caffeine, and nutritional supplements. Each participant arrived at the laboratory after an overnight fast and drinking any type of beverage for 2 h. They were instructed to drink 500 ml of plain water 2 h before all tests to help promote euhydration before the start of each trial. The participants were dressed in T-shirts, shorts, and athletic shoes in all trials. This ensemble was approximately 1.3 kg of the total weight, with a clothing area factor of 1.12, an intrinsic clothing insulation of 0.063 W/(m^2^/°C) or 0.405 clo, and a clothing evaporative resistance of 0.010 W/(m^2^/kPa) (18). All trials were performed outdoors on an old dark-brown brick pavement. All trials were conducted for 1,000 h in August to minimize the effects of solar elevation angle (2) and the time-of-day (19) on thermoregulatory responses. There were no obstructions to sunlight within a 100-m radius during the trials. During exercise, participants faced the sun and received sunlight on the frontal aspect of their body to set the azimuth angle (i.e., the orientation of the body relative to the sun) to 0° (20).

### Exercise protocol

**Figure 1:**
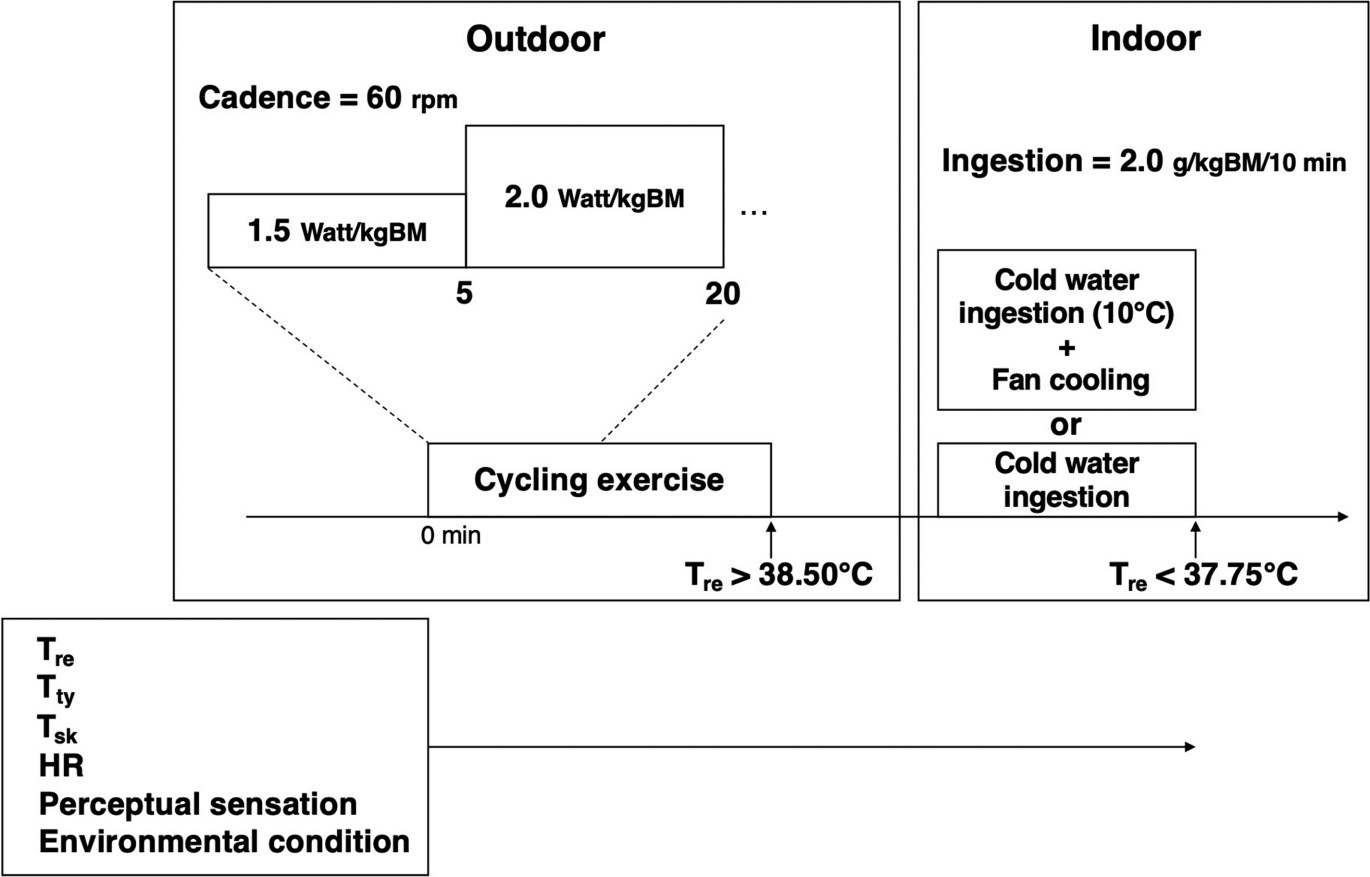
Schematic representation of the experimental protocol. BM, body mass; HR, heart rate; Tty, tympanic temperature; Tre, rectal temperature; Tsk, mean skin temperature.

Given that measuring equipment was attached to participants in laboratory, they went outdoors. All sessions were completed using a cycling ergometer (Monark 828E, Morark, Sweden). The participants rested on the ergometer in seated position for 5 min. Participants repeatedly performed the cycling exercise protocol, which consisted of one set of 5 min at a load of 1.5 watt/kgBM and 15 min at a load of 2.0 watt/kgBM at 60 rpm. This exercise was repeated until the Tre reached 38.50°C (16) (Figure 1).

### Cooling intervention in the post-exercise recovery period

The participants were moved to a warm indoor environment (Table 1) This environment simulated an outdoor recovery in the field that a recreational athlete may experience. During recovery, participants sat on a standard chair. Cold water (10°C) was conventional sports drinks (Aquarius, Japan Coca-Cola, Japan) in simulated usual sporting events. Participants consumed 2.0 g/kgBM of cold water at every 10 min. The fan-cooling jacket was a commercially jacket (Air condition wear, ASICS, Japan) that was employed in a previous study [for detail, see Otani et al. 2021 (13)]. Cooling in both trials continued until Tre reached 37.75°C.

**Table 1:** Environmental conditions at the end of outdoor exercise and during indoor recovery periods.

	Outdoor (Exercise)			Indoor (Recovery)		
	CON	FAN	*P*	CON	FAN	*P*
Global solar radiation (W/m^2^)	1,101 ± 40	1,107 ± 22	0.812			
Mean radiant temperature (°C)	67.9 ± 6.8	68.5 ± 5.5	0.812			
Ambient temperature (°C)	32.9 ± 1.8	33.1 ± 1.8	0.675	30.0 ± 1.7	30.1 ± 1.8	0.905
Relative humidity (%)	50.0 ± 8.0	49.9 ± 8.1	0.812	57.6 ± 6.9	57.2 ± 7.1	1.000
Air velocity (m/sec)	0.8 ± 0.3	0.8 ± 0.3	0.858	0.1 ± 0.1	0.1 ± 0.1	1.000
WBGT (°C)	30.0 ± 0.9	30.2 ± 1.0	0.311	26.6 ± 0.7	26.6 ± 0.8	0.858

Values are means ± SD. CON, no-cooling condition; FAN, wearing fan-cooling jacket; WBGT, wet-buld globe temperature.

### Measurements

All environmental conditions were measured every 15 min, during exercise and recovery periods. Direct and diffuse solar radiation in the horizontal plane were measured at 1.5 m above the ground and recorded using a pyranometer (MS-01, Eko Instruments Co., Ltd., Japan), and global solar radiation was estimated by summing up the values. The horizontal diffuse solar radiation was measured by shielding the pyranometer from direct sunlight. The solar elevation angle was determined from the geographical location (33°30′ North, 130°54′ East). The Ta, RH, black globe temperature and WBGT were measured 1.5 m above the ground and taken using a WBGT meter (WBGT-203A, Kyoto Electronics Industry Co., Ltd., Japan). Air velocity was also measured 1.5 m above the ground using an anemometer (Kestrel 5500, Nielsen–Kellerman, United States).

To evaluate the participants' hydration status, their urine specific gravity was measured using a digital scale (PAL-09S, Atago, Japan) before and after exercise. Nude BM to the nearest 10 g was measured using a weighing scale (BC-316, Tanita, Japan) before and after the trial. Throughout the two trials, Tre was obtained using a disposable rectal thermistor (ITP010-11, Nikkiso-Therm Co., Ltd., Japan) that was self-inserted approximately 150 mm into the rectum. The tympanic temperature (Tty) was obtained using an earplug thermistor (400EP23, Nikkiso-Therm Co., Ltd., Japan) that was self-inserted into the tympanic. Heart rate (HR) monitored continuously *via* telemetry using an HR monitor (H10, Polar, United States) fixed to the chest. Four temperatures (chest, forearm, thigh, and calf) were also recorded *via* iButtons® (Thermocron SL type, KN Laboratory, Japan) affixed using hypoallergenic polyacrylate adhesive tape. Subjective thermal sensation (21) (TS; 9-point scale ranging from 1 = “very cold” to 9 = “very hot”) was recorded at 5-min intervals during recovery.

### Calculations

Tsk was calculated using the following equation (22):

(1) $$\begin{array}{r} \mathrm{Tsk}\;=\;0.3\;\times \;T_{\mathrm{chest}}\;+\;0.3\;\times \;T_{\mathrm{arm}}\;+\;0.2\;\times \;T_{\mathrm{thigh}}\;+\;0.2\;\times \;T_{\mathrm{calf}}, \end{array}$$

where *T*_chest_, *T*_arm_, *T*_thigh_ and *T*_calf_ represent the skin temperature of the chest, upper arm, thigh, and calf (°C), respectively.

*T*_r_ is the mean radiant temperature (°C) calculated using equation 2 below.

The mean radiant temperature (*T*_r_) was calculated using equation 2 (23):

(2) $$T_{r}\;=\;[(T_{g}\;+\;273{)}^{4}\;+\;2.5\;\times \;10^{8}\;\times \;v^{0.6}\left(T_{g}-T_{a}\right){]}^{0.25}-273,$$

where *T*_g_ is the black-globe temperature (°C) and *Ta* is the ambient temperature (°C).

Total sweating volume was calculated as

(3) Totalsweatingvolume=PostBM−PreBM,

Cooling rate in rectal, tympanic, and mean skin temperatures were calculated using the following equation:

(4) $$\mathrm{Cooling}\;\mathrm{rate}\;\mathrm{in}\;\mathrm{Tre}\left({}^{\circ }C/\min\right)=\left(\mathrm{Initial}\;\mathrm{Tre}-37.75\right)/\mathrm{time}\left(\min\right),$$

(5) Cooling rate in Tty or Tsk(∘C/min)=(Initial Tty or Tsk−temperature at the end of recovery)(5)/time in cooling rate in Tre(min),

(6) $$\mathrm{Cooling}\;\mathrm{rate}\;\mathrm{in}\;\mathrm{the}\;\;\mathrm{first}\;20\min\;\mathrm{at}\;\mathrm{each}\;\mathrm{temperature}\left({}^{\circ }C/\min\right)=\left(\mathrm{Initial}\;\mathrm{temperature}-\mathrm{temperature}\;\mathrm{at}\;\mathrm{the}\;\mathrm{end}\;\mathrm{of}\;\mathrm{recovery}\right)/20\left(\min\right).$$

### Statistical analysis

Descriptive data were presented as means ± standard deviations. All statistical computations were performed using the IBM SPSS Statistics 28 software package (SPSS, Inc., United States). The normality of the data and homogeneity of variance between the trials were tested using Shapiro–Wilk's and Levene's tests, respectively. When the result of Shapiro–Wilk's test or Levene's test was less than the significant level, data were analyzed using non-parametric tests. Non-parametric data were analyzed using Friedman's two-way analysis of variance (perceptual sensations). When a significant difference was found, the pairwise comparisons were tested using Wilcoxon's signed-rank test, which was also used to evaluate environmental data. In all other cases, a two-way (trial × time) repeated-measures analysis of variance was performed to compare the data for the different the experimental conditions. Where appropriate, a paired sample *t*-test was then used to identify any changes. The cooling rate, exercise and recovery duration, total sweating volume, and ingestion volume were compared using the dependent samples *t*-test. The level of significance was set at *P* < 0.05. Cohen's d (d) was used as a measure of effect size for paired samples, with 0.2 to <0.6, ≥0.6 to <1.2, ≥1.2 to <2.0, ≥2.0 to <4.0, and ≥4.0 representing small, moderate, large, very large and extremely large treatment effects, respectively (24).

## Results

There were no significant differences in exercise (CON = 36.6 ± 12.5 min, FAN = 34.5 ± 13.5 min, *P* = 0.67, d = 0.16) or recovery (CON = 44.0 ± 5.7 min, FAN = 40.0 ± 11.0 min, *P* = 0.15, d = 0.46) duration between trials. There were no significant differences in indoor and outdoor conditions, heat gain from the sun, or heat loss (*P* > 0.05: Table 1). Comparing the effect of the CON and FAN trials, there were no significantly differences in BM or urine specific gravity (*P* > 0.05). No statistical differences were observed in total sweating (CON = 1.12 ± 0.49 kg, FAN = 1.08 ± 0.50 kg, *P* = 0.69, d = 0.08) or ingestion volume (CON = 532 ± 101 g, FAN = 508 ± 143 g, *P* = 0.56, d = 0.19).

**Figure 2:**
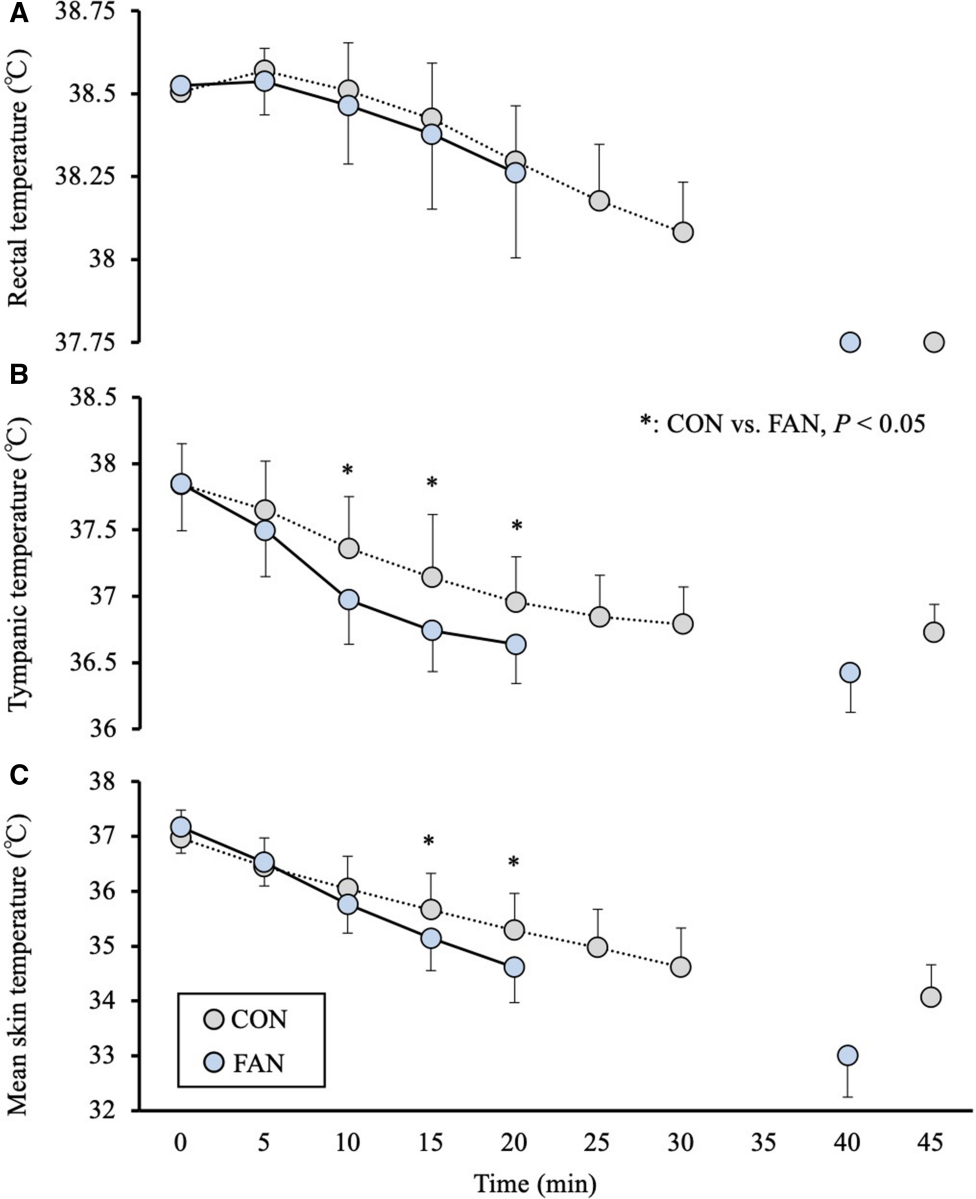
The rectal (**A**), tympanic (**B**) and mean skin temperatures (**C**) under the CON and FAN trials. Time × Trial effect CON vs. FAN: *P < 0.05.

The thermoregulatory responses during the recovery are shown in Figure 2 and Table 2. No significant difference was observed in the Tre between trials during recovery. Tty was lower in the FAN trial from 10 to 20 min than in the CON trial (*P* < 0.05). Tsk was lower in the FAN trial from 15 to 20 min than in the CON trial (*P* < 0.05). Cooling rate in the FAN trial until time to achieve Tre of 37.75°C were greater in Tty (*P *= 0.001, d = 1.12) than the CON trial, albeit no significant differences was observed in Tre (*P *= 0.082, d = 1.05) or Tsk (*P *= 0.136, d = 0.80). Cooling rate in the FAN trial for the first 20 min of recovery were greater in Tty (*P *= 0.002, d = 1.03) and Tsk (*P *= 0.013, d = 1.33) than in the CON trial, albeit no significant differences was observed in Tre (*P *= 0.484, d = 0.85).

**Table 2:** Cooling rate in each body temperature variable during recovery period.

		Time to achieve Tre of 37.75°C	*P*	First 20 min of recovery period	*P*
Rectal tempeature (°C/min)	CON	0.017 ± 0.002	0.082	0.010 ± 0.003	0.484
	FAN	0.021 ± 0.005		0.013 ± 0.004	
Tympanic temperature (°C/min)	CON	0.024 ± 0.010	0.001	0.044 ± 0.017	0.002
	FAN	0.037 ± 0.013*		0.060 ± 0.014*	
Mean skin tempearture (°C/min)	CON	0.061 ± 0.014	0.136	0.084 ± 0.033	0.013
	FAN	0.170 ± 0.193		0.128 ± 0.033*	

Values are means ± SD. CON, no-cooling condition; FAN, wearing fan cooling jacket; Tre, rectal temperature.

* CON vs. FAN (*P* < 0.05).

Perceptual response during recovery was significantly lower in the FAN trial (5 min: 4.7 ± 1.7, 10 min: 3.7 ± 0.9, 15 min: 3.2 ± 0.8, 20 min: 3.2 ± 0.8) than in the CON trial (5 min: 6.6 ± 2.2, 10 min: 5.7 ± 1.8, 15 min: 5.0 ± 1.7, 20 min: 4.4 ± 1.4) from 5 to 20 min in TS (*P* < 0.05, d = 0.97–1.41). There were no significant differences in HR between the two trials.

## Discussion

The main findings of the study are as follows: (1) utilizing a fan-cooling jacket during recovery from exercise in a hot outdoor environment with high solar radiation exposure tended to increase the rate of Tre cooling, but did not significantly differ from cooling rate observed without the fan-cooling jacket. In contrast (2) the utilization of a fan-cooling jacket increased the rate of Tty and Tsk cooling, and (3) decreased subjective thermal strain during the initial periods of recovery.

Although cold water immersion is the most effective technique for rapid body cooling during post-exercise recovery (10), it is not an accessible cooling intervention for most recreational athletes completing outdoor exercise. Fan-cooling jackets are readily available for recreational athletes and have the potential to increase the rate of cooling during recovery, but their efficacy to support recovery from outdoor exercise with high solar radiation exposure is unknown. The decrease rate in Tre during post-exercise recovery in the FAN trial was 0.021 ± 0.002°C/min and did not significantly differ from that in the CON trial. This value is lower than the cooling rate recommended in the treatment of exertional heat stroke (0.15°C/min) as well as the rate obtained using a cooling vest (0.06 ± 0.02°C/min) (25). Differences observed in the above recommended values or the results of previous studies might be due to various factors related to the relationship between Ta and Tsk, skin surface area of the cooling and heat transfer occurring during recovery. Moreover, as the temperature gradient between ambient and skin increases, conductive heat loss also increases. In this study, the ambient temperature during the recovery period, which was assumed to take place in a shaded environment at an indoor temperature of approximately 30°C, was lower than Tsk. The rate of decrease in Tre was probably lower because the recovery period took place in a warm environment rather than an air-conditioned environment. Barwood et al. (15) noted that fan cooling jackets, compared with whole-body fanning, have less cooling effect since they cover smaller body surface areas. In addition, the abovementioned previous studies showed that using whole body fanning during post-exercise recovery significantly reduced Tre, compared with not using it, while no difference was observed using fan cooling jackets. This may indicate that the cooling power of simple cooling devices, such as fan cooling jackets, may not be sufficient to effectively reduce Tre during recovery after exercise in a hot outdoor environment under solar radiation. Among recreational athletes participating in community level sporting events, who do not have access to cold water immersion as a cooling mechanism, the utilization of a jacket to reduce core temperature is not an appropriate method to alleviate heat stroke. Therefore, recreational athletes may be needed further cooling option such as internal cooling (e.g., ice slurry ingestion) (26).

Tsk showed a significantly higher cooling rate in the FAN trial than in the CON trial during the first 20 min of recovery, but no difference was observed during the recovery period as a whole. The use of fan airflow for body cooling promoted heat dissipation by accelerating evaporation of the sweat secreted onto the skin surface through increased air movement (27, 28). Although the local sweating rate was not measured in this study, it is speculated that one of the possible reasons for the reduction in heat dissipation may be the lack of sweat distribution over the skin surface during the latter half of recovery compared to the first 20 min (i.e., high wetness). It is possible that if this study had been permitted to wipe their skin with a towel, we would have obtained different results from this study. In body cooling with fan-cooling jackets, the air, sucked in by fans, creates a layer between the skin and clothing, which triggers heat transfer *via* convection (29). Although temperature inside clothing was not measured in this study, it was hypothesized that the transfer of hot and humid air through clothing contributed to the reduction in Tsk. The cooling rate of Tsk throughout the whole recovery period varied greatly among individuals. Local sweating rates must also be considered in future studies as this may indicate that sweat secretion was also occurred in some of the study subjects during the latter half of recovery. Additionally, it is noteworthy that summertime in Japan is hot and humid, and sweat becomes widely distributed over the skin surface without evaporating. In this study, we demonstrated that body cooling recovery using a fan-cooling jacket is an effective strategy for promoting heat dissipation and reducing high Tsk caused by exposure to solar radiation. In addition, the core-to-skin temperature gradient was wider with a lower Tsk in the FAN trial that suggested a physiological benefit, although Tre was unchanged.

The cooling rate in the Tty during recovery was significantly higher in the FAN trial than in the CON trial. These results are in line with that of previous study that used tympanic temperature as an estimated index of core temperature and fan-cooling jackets as a cooling technique between exercise bouts and during short recovery periods (13). In terms of Tc measurement, the tympanic site showed lower temperatures and faster responses compared to the rectal site (30). The higher cooling rate in the Tty observed in this study may be due to the fact that the effect of the fan-cooling jacket was reflected earlier in the tympanic area near the tympanic membrane. In addition, the cooling effect and fall of temperature in the tympanic and cervical region, induced by the airflow of fan-cooling jacket circulating in the front and back of the neck (13), may be the result of heat transfer *via* conduction from that area.

The results of this study highlight that wearing a fan-cooling jacket during recovery, after exposure to high solar radiation during exercise in a hot outdoor environment, is an effective method to promote reduction of tympanic temperature and perceptual thermal strain, but not rectal temperature or HR. Finally, most studies on fan-cooling jackets and airflow cooling have been conducted in a laboratory setting, and the lack of outdoor experimentation under conditions of high radiant heat has been a major issue in previous studies (13, 27). The findings of this study may provide better alternatives for improving subjective indicators after exposure to high radiant heat, not only for athletes but also for recreational athletes who do not own specialized equipment.

## Conclusion

This study indicates that a fan-cooling jacket in post-exercise recovery did not enhance increase the rate of decrease in Tre in the FAN trial. However, the fan-cooling jacket resulted in significantly greater rate Tty or Tsk in the first 20 min of recovery than cold water ingestion. These results suggested that body cooling recovery with a fan-jacket in addition to cold water ingestion may be an effective strategy for reducing skin surface, and subjective sensation increased by high radiant heat during exercise, but it may be difficult to induce a decrease in Tc. Thus, the study will help in the better management of hyperthermia in athletes and recreational athletes.

## Data Availability

The raw data supporting the conclusions of this article will be made available by the authors, without undue reservation.

## References

[1] Budd GM. Wet-bulb globe temperature (WBGT)–its history and its limitations. J Sci Med Sport. (2008) 11(1):20–32. 10.1016/j.jsams.2007.07.003 17765661

[2] Otani H Goto T Goto H Shirato M. Time-of-day effects of exposure to solar radiation on thermoregulation during outdoor exercise in the heat. Chronobiol Int. (2017) 34(9):1224–38. 10.1080/07420528.2017.1358735 28910548

[3] O’Connor FK Stern SE Doering TM Minett GM Reaburn PR Bartlett JD Effect of individual environmental heat-stress variables on training and recovery in professional team sport. Int J Sports Physiol Perform. (2020) 15(10):1393–9. 10.1123/ijspp.2019-0837 32590345

[4] Galloway SD Maughan RJ. Effects of ambient temperature on the capacity to perform prolonged cycle exercise in man. Med Sci Sports Exerc. (1997) 29(9):1240–9. 10.1097/00005768-199709000-00018 9309637

[5] Naito T Saito T Muraishi K Takahashi H. Comparison of the effects of high and low levels of solar radiations on exercise capacity in hot outdoor environments. J Sports Med Phys Fitness. (2023) 63(1):42–52. 10.23736/S0022-4707.22.13627-3 35415996

[6] Otani H Kaya M Tamaki A Goto H Maughan RJ. Exposure to high solar radiation reduces self-regulated exercise intensity in the heat outdoors. Physiol Behav. (2019) 199:191–9. 10.1016/j.physbeh.2018.11.029 30471385

[7] O’Connor FK Doering TM Minett GM Reaburn PR Bartlett JD Coffey VG. Effect of divergent solar radiation exposure with outdoor versus indoor training in the heat: implications for performance. J Strength Cond Res. (2022) 36(6):1622–8. 10.1519/JSC.0000000000003706 32658031

[8] Sawka MN Cheuvront SN Kenefick RW. High skin temperature and hypohydration impair aerobic performance. Exp Physiol. (2012) 97(3):327–32. 10.1113/expphysiol.2011.061002 22143882

[9] Seeley AD Sherman RA. An ice vest, but not single-hand cooling, is effective at reducing thermo-physiological strain during exercise recovery in the heat. Front Sports Act Living. (2021) 3:660910. 10.3389/fspor.2021.660910 33997780 PMC8117958

[10] Casa DJ DeMartini JK Bergeron MF Csillan D Eichner ER Lopez RM National athletic trainers’ association position statement: exertional heat illnesses. J Athl Train. (2015) 50(9):986–1000. 10.4085/1062-6050-50.9.07 26381473 PMC4639891

[11] Douma MJ Aves T Allan KS Bendall JC Berry DC Chang WT First aid cooling techniques for heat stroke and exertional hyperthermia: a systematic review and meta-analysis. Resuscitation. (2020) 148:173–90. 10.1016/j.resuscitation.2020.01.007 31981710

[12] Zhang Y Davis JK Casa DJ Bishop PA. Optimizing cold water immersion for exercise-induced hyperthermia: a meta-analysis. Med Sci Sports Exerc. (2015) 47(11):2464–72. 10.1249/MSS.0000000000000693 25910052

[13] Otani H Fukuda M Tagawa T. Cooling between exercise bouts and post-exercise with the fan cooling jacket on thermal strain in hot-humid environments. Front Physiol. (2021) 12:640400. 10.3389/fphys.2021.640400 33664676 PMC7920971

[14] Hashimoto K Horie S Nagano C Hibino H Mori K Fukuzawa K A fan-attached jacket worn in an environment exceeding body temperature suppresses an increase in core temperature. Sci Rep. (2021) 11(1):21269. 10.1038/s41598-021-00655-2 34711896 PMC8553827

[15] Barwood MJ Davey S House JR Tipton MJ. Post-exercise cooling techniques in hot, humid conditions. Eur J Appl Physiol. (2009) 107(4):385–96. 10.1007/s00421-009-1135-1 19649650

[16] Mori K Nagano C Fukuzawa K Hoshuyama N Tanaka R Nishi K Mitigation of heat strain by wearing a long-sleeve fan-attached jacket in a hot or humid environment. J Occup Health. (2022) 64(1):e12323. 10.1002/1348-9585.12323 35384178 PMC9176717

[17] Wang F Chow CS-W Zheng Q Ke Y Yang B Zheng X On the use of personal cooling suits to mitigate heat strain of mascot actors in a hot and humid environment. Energy Build. (2019) 205:109561. 10.1016/j.enbuild.2019.109561

[18] Zuo J McCullough EA. Heat transfer characteristics of sports apparel. J ASTM Int. (2004) 1(10):10. 10.1520/JAI12143

[19] Otani H Kaya M Tamaki A Goto H Goto T Shirato M. Diurnal effects of prior heat stress exposure on sprint and endurance exercise capacity in the heat. Chronobiol Int. (2018) 35(7):982–95. 10.1080/07420528.2018.1448855 29561175

[20] Underwood CR Ward EJ. The solar radiation area of man. Ergonomics. (1966) 9(2):155–68. 10.1080/00140136608964361 5930131

[21] ISO. Ergonomics of the physical environment—subjective judgement scales for assessing physical environments. ISO 10551. (2019):28.

[22] Ramanathan NL. A new weighting system for mean surface temperature of the human body. J Appl Physiol. (1964) 19:531–3. 10.1152/jappl.1964.19.3.531 14173555

[23] ISO. Ergonomics of the thermal environment—instruments for measuring physical quantities. ISO 7226. (1998).

[24] Hopkins WG Marshall SW Batterham AM Hanin J. Progressive statistics for studies in sports medicine and exercise science. Med Sci Sports Exerc. (2009) 41(1):3–13. 10.1249/MSS.0b013e31818cb278 19092709

[25] Hosokawa Y Belval LN Adams WM Vandermark LW Casa DJ. Chemically activated cooling vest’s effect on cooling rate following exercise-induced hyperthermia: a randomized counter-balanced crossover study. Medicina. (2020) 56(10):539. 10.3390/medicina56100539 PMC7602153 33066469

[26] Naito T Haramura M Muraishi K Yamazaki M Takahashi H. Impact of ice slurry ingestion during break-times on repeated-sprint exercise in the heat. Sports Med Int Open. (2020) 4(2):E45–E52. 10.1055/a-1139-1761 32395608 PMC7205515

[27] Lynch GP Periard JD Pluim BM Brotherhood JR Jay O. Optimal cooling strategies for players in Australian tennis open conditions. J Sci Med Sport. (2018) 21(3):232–7. 10.1016/j.jsams.2017.05.017 28647283

[28] Havenith G Holmér I den Hartog EA Parsons KC. Clothing evaporative heat resistance—proposal for improved representation in standards and models. Ann Occup Hyg. (1999) 43(5):339–46. 10.1016/S0003-4878(99)00052-6 10481633

[29] Suzuki E Kashimura O Takahashi H Minami K Nakai S. Effects of air circulation in clothing inside on thermoregulatory responses at farming under heat environment conditions. Jpn J Biometeor. (2012) 49(2):83–92.

[30] Huggins R Glaviano N Negishi N Casa DJ Hertel J. Comparison of rectal and aural core body temperature thermometry in hyperthermic, exercising individuals: a meta-analysis. J Athl Train. (2012) 47(3):329–38. 10.4085/1062-6050-47.3.09 22892415 PMC3392164

